# CRISPR/Cas9-mediated gene editing to confer turnip mosaic virus (TuMV) resistance in Chinese cabbage (*Brassica rapa*)

**DOI:** 10.1093/hr/uhad078

**Published:** 2023-04-21

**Authors:** Ye-Rin Lee, Muhammad Irfan Siddique, Do-Sun Kim, Eun Su Lee, Koeun Han, Sang-Gyu Kim, Hye-Eun Lee

**Affiliations:** Vegetable Research Division, National Institute of Horticultural and Herbal Science, Rural Development Administration, Wanju, 55365, Republic of Korea; Department of Horticultural Sciences, North Carolina State University Mountain Horticultural Crops Research, Extension Center 455 Research Drive, Mills River, NC 28759, USA; Vegetable Research Division, National Institute of Horticultural and Herbal Science, Rural Development Administration, Wanju, 55365, Republic of Korea; Vegetable Research Division, National Institute of Horticultural and Herbal Science, Rural Development Administration, Wanju, 55365, Republic of Korea; Vegetable Research Division, National Institute of Horticultural and Herbal Science, Rural Development Administration, Wanju, 55365, Republic of Korea; Department of Biological Sciences, Korea Advanced Institute for Science and Technology, Daejeon, 34141, Republic of Korea; Vegetable Research Division, National Institute of Horticultural and Herbal Science, Rural Development Administration, Wanju, 55365, Republic of Korea

## Abstract

Genome editing approaches, particularly the CRISPR/Cas9 technology, are becoming state-of-the-art for trait development in numerous breeding programs. Significant advances in improving plant traits are enabled by this influential tool, especially for disease resistance, compared to traditional breeding. One of the potyviruses, the turnip mosaic virus (TuMV), is the most widespread and damaging virus that infects *Brassica* spp. worldwide. We generated the targeted mutation at the *eIF(iso)4E* gene in the TuMV-susceptible cultivar “Seoul” using CRISPR/Cas9 to develop TuMV-resistant Chinese cabbage. We detected several heritable indel mutations in the edited T_0_ plants and developed T_1_ through generational progression. It was indicated in the sequence analysis of the *eIF(iso)4E*-edited T_1_ plants that the mutations were transferred to succeeding generations. These edited T_1_ plants conferred resistance to TuMV. It was shown with ELISA analysis the lack of accumulation of viral particles. Furthermore, we found a strong negative correlation (*r = −0.938*) between TuMV resistance and the genome editing frequency of *eIF(iso)4E*. Consequently, it was revealed in this study that CRISPR/Cas9 technique can expedite the breeding process to improve traits in Chinese cabbage plants.

## Introduction

Plant viruses pose a severe threat to global crop yield. There are many approaches to developing virus-resistant cultivars in crop plants. Among them, one common approach is traditional plant breeding by incorporating virus resistance genes from wild relatives into elite cultivars [1–4]. A recent alternative approach is genome editing, which instantly allows the importation of alleles mediating resistance into crop plants, preventing long and laborious backcrosses in traditional breeding [1, 5]. The genome-edited crops can be transgenic free [5, 6], and they probably would be classified as non-genetically modified food crops [7]. Thus, the genome editing technique may be a publicly acceptable advanced breeding method and opens up a new avenue for developing virus-resistant cultivars.

CRISPR/Cas9 (clustered regularly interspersed palindromic repeats/CRISPR-associated protein 9) is a site-specific genome editing approach originating from the adaptive immune system of *Streptococcus pyogenes* against bacteriophages [8]. CRISPR/Cas9 technique was first reported in 2012, and ever since, it has turned out to be the most frequently used technology among plant biologists due to its low cost, simplicity, and considerably smaller timespan for construct formulation compared to alternative genome editing techniques [9]. Accurate genome editing of host factors can be used to develop recessive genetic resistance in plants against viral diseases [10]. Nevertheless, the implementation of CRISPR/Cas9 technology has not been frequently employed to deploy genetic resistance against pathogens, except for a several reports [1, 5, 11–15].

Potyviridae, the most prominent family of plant RNA viruses, account for around 30% of reported plant viruses, causing many significant injuries to crop plants [16, 17]. Especially, among the potyviruses, such as TuMV has been reported to severely threaten Chinese cabbage (*Brassica rapa*) crops [18]. Interestingly, plant RNA viruses entail host factors to sustain their life cycle [19, 20]. Several genes conferring resistance to viruses are recessive (Kang et al. 2005a; [21]), including eukaryotic translation initiation factor *(eIF)* genes, for example, eukaryotic translation initiation factor 4E (*eIF4E*), eukaryotic translation initiation factor 4G (*eIF4G*), and eukaryotic translation initiation factor *(iso)4E* (*eIF(iso)4E*) [19, 20]. Different plant species contain different numbers of *eIF4E* family members. In *Arabidopsis*, *eIF(iso)4E* is a significant host factor in TuMV resistance [22–26]. Following this, the *Brassica eIF(iso)4E* gene has been reported to be tightly associated with the *Brassica* recessive resistance genes *retr02* and *trs* [27, 28]. It was purposed by these results that the *eIF(iso)4E* gene could be a suitable target for the genome editing of TuMV resistance in *Brassica* species.

The CRISPR/Cas9 genome technology has been applied for developing potyvirus-resistant crops [1, 5, 12, 14]. For example, the genome editing of the gene *eIF(iso)4E* in *A. thaliana* conferred resistance against the turnip mosaic virus (TuMV) [14]. It was revealed in another research that the CRISPR/Cas9-mediated genome editing of *eIF4E* in cucumber resulted in broad-spectrum viral resistance to numerous plant viruses, such as cucumber vein yellowing virus (CVYV), papaya ringspot mosaic virus-w (PRSV-W), and zucchini yellow mosaic virus (ZYMV) [1]. Likewise, resistance against the rice tungro spherical virus was conferred by a novel allelic mutation of rice *eIF4G*, developed using the CRISPR/Cas9 genome editing approach [12]. Recently, editing the *eIF4E* in tomatoes also resulted in resistant tomatoes against the pepper mottle virus (PepMoV) [5].

We developed TuMV-resistant Chinese cabbage (*B. rapa*) by mutating *eIF(iso)4E* in the Chinese cabbage cultivar “Seoul” using the CRISPR/Cas9 approach and *Agrobacterium*-mediated transformation in this study. We generated the T_0_ transgenic plants carrying *eIF(iso)4E* mutations and produced T_1_ progeny. We confirmed the indel frequency within the target regions of the edited mutant T_0_ and T_1_ plants and examined the resistance of T_1_ plants to TuMV. Overall, this study provides a protocol for analyzing and interpreting Chinese cabbage CRISPR/Cas9 mutants and improving the targeted traits in crops using genome editing technology.

## Results

### Transformation and generation of Chinese cabbage

We inserted the CRISPR/Cas9 constructs (pECO101-hyg-Cas9-*eIF(iso)4E* vector) carrying the corresponding sgRNAs into the *B. rapa* cultivar, “Seoul,” through *Agrobacterium*-mediated transformation to develop Chinese cabbage plants in which the *eIF(iso)4E* gene has been edited (Fig. S1). We cultured the shoot in a shooting medium to increase the elongation of the shoot induced by the callus. The regenerated shoot was relocated to the rooting medium to promote root formation (Fig. S1). PCR analysis was performed using Cas9-specific primers to confirm and select a transgenic entity harboring transfer DNA (T-DNA) in a regenerated plant (Table 2). Thirteen of the 15 regenerated plants confirmed the Cas9 transgenes by showing PCR products with the expected amplicon sizes (600 bp). The transformation efficiency was 86.7% (Fig. 2A).

### Confirmation of CRISPR/Cas9-induced T_0_ plant to gene editing efficiency

We performed the targeted deep-sequencing using the PCR product to ensure whether the CRISPR/Cas9 induced mutations in the *eIF(iso)4E* gene. Unfortunately, we confirmed that only one sgRNA out of three sgRNAs constructed into the pECO101-hyg-Cas9-*eIF(iso)4E* vector sgRNA3 targeting three *eIF(iso)4E* genes had high editing efficiency. Among the *eIF(iso)4E*-related genes, we was confirmed for gene correction only for Bra035393, a TuMV recessive resistance-related gene reported by Kim et al. [27]. The T_0_ edited plants #4, #6, #7, and #12 were confirmed by deep sequence analysis that had high gene editing efficiency among the selected plants. Four types of sequence variations were observed: one nucleotide (A, T, or C) insertion (+1) into the target region or twenty eight nucleotide deletion (−28), including the protospacer adjacent motif (PAM) sequence (Fig. 2B). The total indel frequencies were 47.4% for #4, 47.2% for #6, 62.8% for #7, and 49.1% for #12 T_0_ edited plants. The primary indel frequencies were 21.1%, 43.6%, 52.8%, and 20.2%, respectively (Fig. 2B). No homozygous mutant lines were found among the T_0_ edited plants.

### Identification of Indel pattern and frequency in T_1_ edited plants

For propagation to T_1_ seeds, #6 and #7 T_0_ edited plants, which consisted of one significant indel pattern among the four T_0_ edited plants (#4, #6, #7, and #12), were self-fertilized (Fig. 2B). We extracted gDNA from the leaf samples of the #6 and #7 T_1_ edited plants and verified the existence of transgene by PCR with transgene-specific primers (Table 2). Deep sequence analysis was performed on the T_1_-edited plants to measure the editing efficacy (Fig. S2A and B). Although the T_0_-edited plants had one significant indel pattern, and the generational progression was achieved using self-fertilization, the T_1_-edited plants showed various new indel patterns. We divided the different indel patterns into three groups: (i) single, (ii) double, and (iii) mosaic (Fig. 3). In the group having a single indel pattern, #6 and #7 T_1_ edited plants showed the same indel pattern as T_0_ edited plants. In addition, #7–53, #7–62, 7–66, and #7–72 showed another nucleotide (A) insertions, and #7–79 showed PAM site deletions (Fig. 3A). In the group having a double indel pattern, one of the two indel patterns showed the same pattern as T_0_ plants. In the case of #6–41 and #6–42, low mutant frequency, but a different nucleotide (A) was inserted. Plant #6–79 had two deletions that were not observed in T_0_ (Fig. 3B). In #7–73 and 7–78, PAM sites were deleted, and in #7–74 and #7–84, different nucleotides (A) were inserted (Fig. 3B). In the group having mosaic indel pattern, a nucleotide different from the significant pattern of T_0_ was inserted in most T_1_ edited plants (Fig. 3C). The highest total indel frequency was #6–62, #6–64, and #7–79, and were 99.8%, 99.8%, and 100, respectively (Fig. 3). The lowest total indel frequency was 55.0% for #6–76 and 6.6% for #7–81.

### TuMV resistance in *eIF(iso)4E* T_1_ edited plants

We inoculated TuMV into the single and double indel pattern T_1_ edited #6 and #7 T_0_ plants and wild-type control (Fig S3A and B) to examine whether these CRISPR/Cas9-derived *eIF(iso)4E* mutants of Chinese cabbage are resistant to TuMV. TuMV caused several leaf symptoms in infested plants, for example, conventional systemic mosaic, leaf curling, chlorotic lesions, chlorotic mottling, vein clearing, necrotic lesions, and stunted plant growth. The wild-type plant displayed distinctive TuMV symptoms as early as seven days post inoculation (DPI), containing vein clearing and several small chlorotic mottling in the leaves (Fig. S3C). We performed the DAS-ELISA analysis using the inoculated leaves of #6 and #7 T_1_ edited plants after 21 DPI (Fig. 4) to verify virus infection. We detected considerable amounts of virus coat protein accumulation in the leaves of the susceptible wild-type control. However, coat protein hardly accumulated in the systemic leaves of any edited mutant plants, validating that the *eIF(iso)4E*-edited mutant plants were resistant to TuMV (Fig. 4).

**Table 1:** BLAST and GenBank accession number of the *eIF(iso)4E* gene in *Brassica rapa*

**Brassica ID** ^**a**^	**Position of the gene (bp)**		**Strand**	**Gene description** ^**b**^	**GenBank accession number** ^**c**^
	**Start**	**End**			
*Bra035531*	8 297 320	8 298 447	+	*BraA.eIF(iso)4E.c*	HM131211.1
*Bra039484*	8 702 297	8 703 577	−	*BraA.eIF(iso)4E.b*	HM131210.1
*Bra035393*	555 722	557 040	+	*BraA.eIF(iso)4E.a*	HM131209.1

a The orthologs found in BLAST searches of the Brassica database (BRAD)

b The *eIF(iso)4E* copies reported by Jenner et al. [35]

c The *eIF(iso)4E* copies found in NCBI GenBank

**Figure 1:**
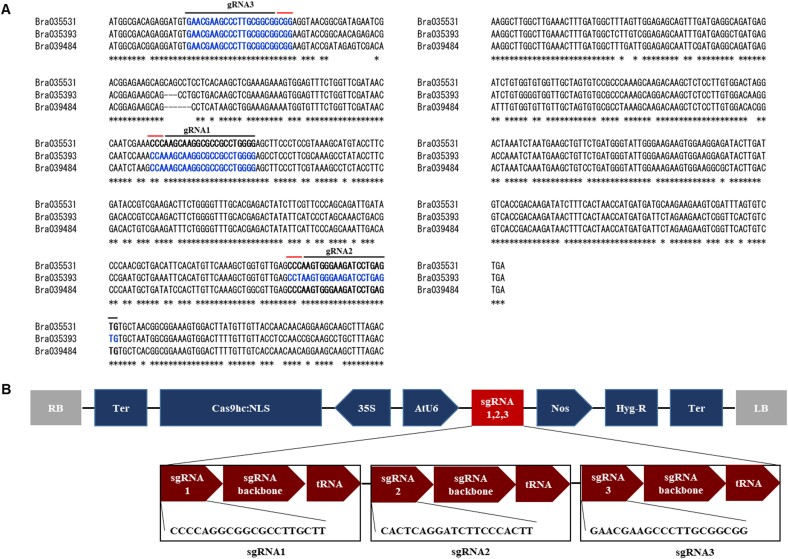
Schematic representation of the CRISPR/Cas9 vector constructs and characteristics information of sgRNA target sites. A) Nucleotide sequence of the *eIF(iso)4E* gene alignment in Chinese cabbage. Target sequences are in blue and black upper-lined, and the protospacer adjacent motif (PAM) sequences (NGG) are in red upper-line. B) Diagram of the CRISPR/Cas9 vector constructs and target sites within the gRNA of *eIF(iso)4E* genes. RB, right border; Ter, terminator; 35S, CaMV 35S promoter; Nos, Nos promoter; Hyg-R, hygromycin resistance gene; LB, left border. The sgRNA structures are represented in each box.

### Differences in TuMV resistance according to gene editing efficiency

Next, we measured the correlation between the total indel frequency of the T_1_ edited plants and ELISA scores to clarify the role of *eIF(iso)4E* in TuMV resistance. The #6–57 plant, which showed the highest susceptibility when observed at seven DPI (not shown data), had 8.5% of the total indel frequency and 2.0485 of the ELISA score at 21 DPI as a susceptible plant. The #6–62 and #6–76 edited plants with 99.8% and 55.0% of total indel frequency showed 0.1094 and 0.7593 ELISA scores, respectively. It was revealed in the correlation analysis that the total indel frequency had a high negative correlation (*r = −0.938*) with the ELISA score (Fig. 5), confirming that *eIF(iso)4E* was a significant host factor in TuMV resistance.

**Table 2:** List of primers used in the current study.

Name	Primer sequence (5′ to 3′)	Amplicon size (bp)	Purpose
Hyg_F	GCGAAGAATCTCGTGCTTTC	209	Mutation detection
Hyg_R	CAACGTGACACCCTGTGAAC		
MY5 - AtU6 _F	AAGAAGAGAAGCAGGCCC	600	Transgene confirmation
YM20 R - eIF4E-gRNA3_R	AAACCCGCCGCAAGGGCTTCGTTC		

**Figure 2:**
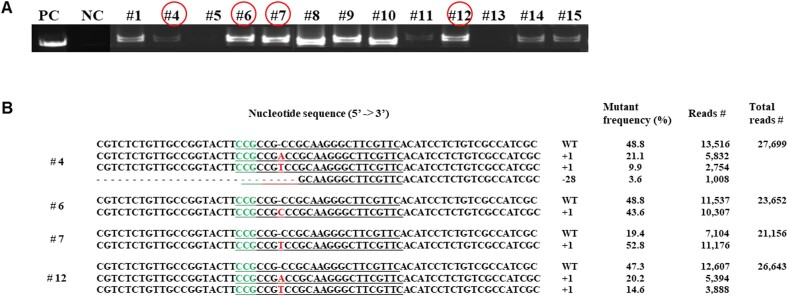
Confirmation of transformation by PCR analysis and indel frequency and patterns for recessive TuMV resistance gene (*Bra035393*) in T_0_ transgenic plants. A) PCR analysis to confirm the transgenes encoding Cas9 and gRNAs in T_0_ transgenic plants. PC, positive control (Cas9 plasmid); NC, negative control (non-transgenic plant). B) DNA sequencing of mutated T_0_ transgenic plants. The mutant frequency was calculated by dividing the number of reads containing indels at the target site (Reads #) by the total sequencing reads. Target sequences are underlined. The protospacer adjacent motif (PAM) sequences (CGG) are in green. Insertions and deletions are represented by red font and red hyphens, respectively. WT, wild-type; +, insertion; −, deletion.

### Phenotype evaluation of TuMV resistance edited plants

We confirmed TuMV symptoms between edited and wild type (WT) plants 30 DAI (days after inoculation) to evaluate the phenotype (Fig. 6A). Edited plants that showed resistance to TuMV and small symptoms of TuMV by the total indel frequency, but there were no experienced any major issues with growth and development (Fig. 6B). While, WT plants exhibited mosaic symptoms, which are typical of TuMV, throughout their leaves, resulting in poor conditions for growth and development (Fig. 6B).

We transplanted the inoculated plants into pots and grew them with the non-inoculated plant (mock) in an LMO glass house to evaluate the resistance of the wild type (WT) and edited plants to TuMV. After 30 days of planting in a pots, phenotypic evaluation was performed with the naked eye. Likewise, all the edited plants were resistant to TuMV, the phenotypic difference was observed according to the total indel frequency. Plant #6–62 with a high total indel frequency (99.8%) showed no phenotypic difference from the mock, but in the case of plant #6–76 with a medium total indel frequency (55.0%), there was no problem in growth. Still, mosaic symptoms, one of the viral symptoms, appeared in the leaves. Also, mosaic symptoms appeared in newly emerging shoots (Fig. 7). Most of the WT plants died, and even if they survived as mature plants, mosaic symptoms were severe and there was a noticeable problem in growth and development (Fig. 7).

**Figure 3:**
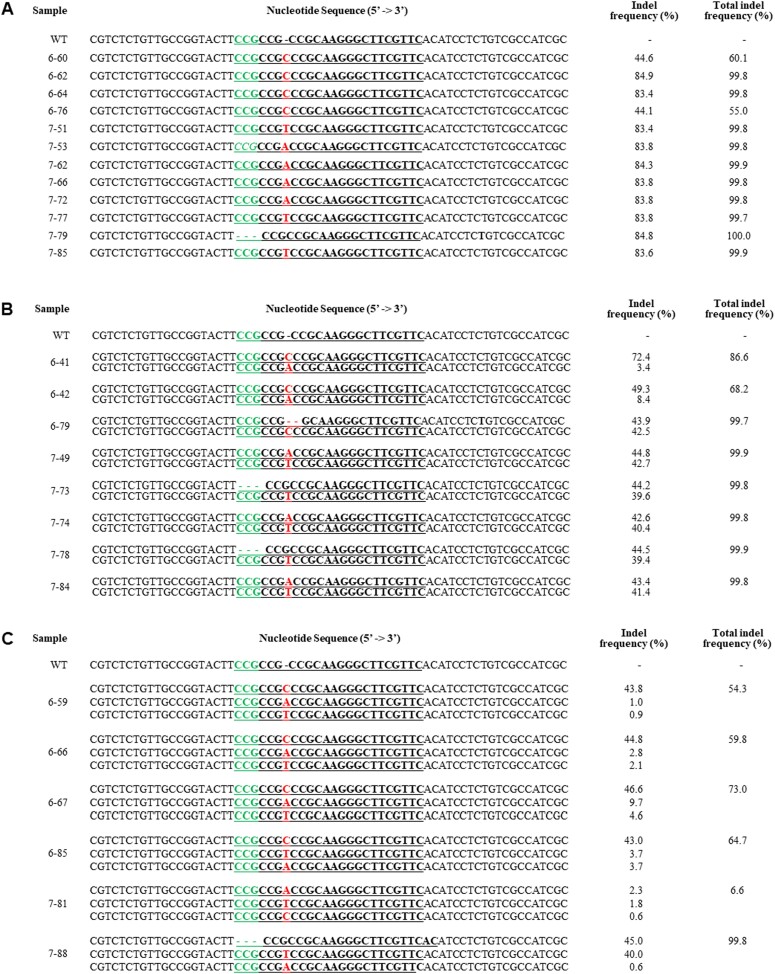
Inheritability of the *eIF(iso)4E*–mutated (*Bra035393*) T_1_ edited plants derived from #6 and #7 transgenic plants. Indel mutation patterns in T_1_ progenies of T_0_ plants. A) single; B) double; C) mosaic. The indel frequency was calculated by dividing the number of reads containing indels at the target site (Reads #) by the total sequencing reads. The target sequence is underlined. The protospacer adjacent motif (PAM) sequences (CGG) are in green. Insertions and deletions are represented by red font and hyphens, respectively. WT means wild-type.

**Figure 4:**
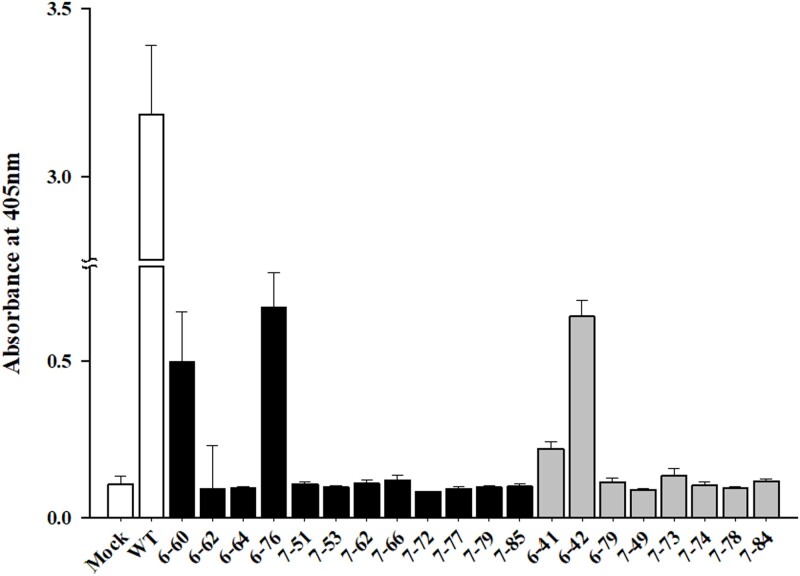
Resistance analysis of *eIF(iso)4E*-mutated (*Bra035393*) plants challenged with TuMV by ELISA analysis 21 days post inoculation. Analysis of TuMV coat protein accumulation in inoculated and systemic leaves of single (black bar) and double (gray bar) indel pattern #6 and #7 T_1_ edited plants by DAS-ELISA. The mutation patterns were described in Fig. 3. Error bars indicate the mean values of replicates ± SD.

## Discussion

Food security is one of the major problems confronting the modern world due to the constantly increasing global population. According to estimates, global food production should be doubled in the next few decades to cope with ever-increasing consumer requirements [14, 29]. It has been appraised that viral infection in food crops ensues around 10–15% yield loss annually worldwide (Kang et al. 2005a; [30]). Extenuation of these losses by incorporating viral resistance into existing elite verities is a promising approach to achieving global food production goals. Exploiting genetic resistance in crops is the most endurable strategy to manage virus infection (Kang et al. 2005a; [31, 32]). Modern innovative plant biotechnology and molecular breeding approaches can assist as preferable substitutes for traditional breeding. A principal objective of plant biotechnology is to enhance crop production and quality in an endurable manner more expeditiously than conventional breeding. Several host genes connected with virus resistance have been reported in previous research on plant-virus interactions (Kang et al. 2005a; Gomez et al. 2009; [2]).

Furthermore, transgenic methods to introgress resistance in various crops have been effectively used against several viruses in the last couple of decades [33]. Nonetheless, complications encountered in regulatory provisions and socio-economic issues, such as the public unacceptability of transgenic crops, are significant constraints to widely implementing these biotechnological approaches. In contrast, genome-edited crops can be transgenic free [5, 6, 34]. They would probably be classified as non-genetically modified food crops [7].

Since recessive resistance genes are deemed to provide more durable resistance compared with dominant resistance genes in the present study, we focused on developing CRISPR/Cas9-edited mutants to establish virus resistance in Chinese cabbage. We targeted the *eIF(iso)4E* gene using the CRISPR/Cas9 system to achieve this, and developed TuMV resistance to potyvirus in *B. rapa* Chinese cabbage, cultivar “Seoul,” through *Agrobacterium*-mediated transformation.

In *Arabidopsis*, mutations in *eIF(iso)4E* resulted in TuMV resistance, and there is only a single copy gene [24]. In contrast, *B. rapa* has multiple copies of the *eIF(iso)4E* gene [27, 35], and each gene was sequenced using cloned genomic DNA by Kim et al. [27]. We found three copies related to *eIF(iso)4E* in *B. rapa* and confirmed the sequence in NCBI to distinguish the gene for each copy (Table 1). In several previous studies [27, 28], the TuMV-related recessive gene was reported as Bra035393 (*BraA.eIF(iso)4E.a*), whereas *Bra035531* (*BraA.eIF(iso)4E.c*), it has been reported that there is no specific variation between susceptible and resistant sequences [27]. Therefore, we first performed the gene editing analysis focusing on *Bra035393* gene, and as a result of this study, edited-plant with resistance to TuMV obtained. Since we targeted TuMV resistance, we performed gene editing analysis targeting only *Bra035393* by referring to the results of previous studies in this study, but we plan to additionally perform gene editing analysis for the other two copies of the gene (*Bra035531* and *Bra039484*).

The *eIF(iso)4E* edited mutant plants showed indels eventuated at numerous locations within the sgRNA target site and, in some cases, near the sgRNA target region (Fig. 2 and 3). In our results, the *eIF(iso)4E*-targeted mutant plants in the T_0_ generation were double indel patterns. However, we observed a mixture of mutant variations, including single, double, and mosaic indel patterns in T_1_-edited plants. The mosaicism has been reported previously in various edited plants [1, 5, 14, 34, 36–40]. The mosaic indel patterns of the T_1_ edited plants might be because the Cas9 protein and sgRNA are highly expressed in some edited plants. Moreover, additional alleles produced from lately-emerging chimeric tissues could also be unnoticed in T_0_ plants, consequential ensuing in different flowers exhibiting different alleles [36]. Because of this complexity, we advanced the T_0_ plants with a simple primary indel pattern for the gene of interest in the next generations (Fig. 2). It was confirmed in further sequence analysis that the indel frequency in T_1_ edited plants (Fig. 3), suggesting that their mutant status was stably transmitted to their progenies as generation progressed [1, 5].

**Figure 5:**
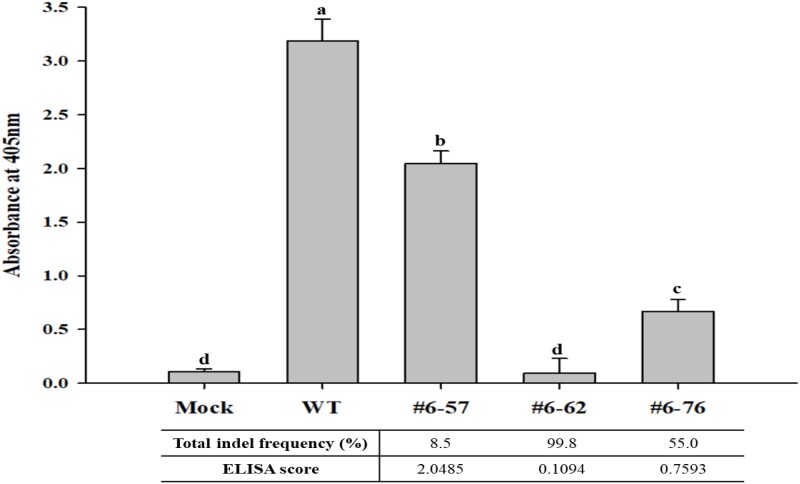
Correlation between TuMV resistance and gene editing frequency. Gene editing frequency means the total indel frequency for each edited plant. ELISA analysis was conducted on samples collected 21 days post inoculation. Error bars indicate the mean values of replicates ± SD. Different letters (a, b, c, and d) denote significant differences (*P < 0.05*) among edited plants, according to Duncan’s test.

**Figure 6:**
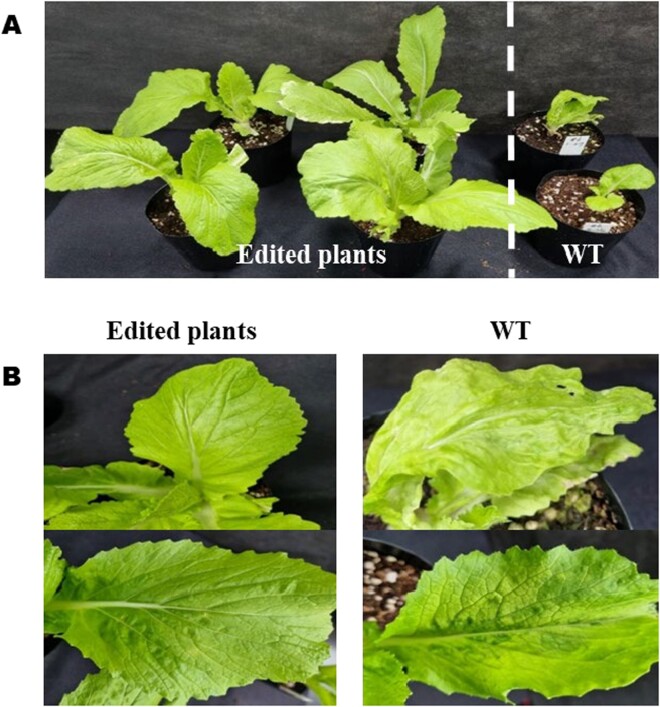
Phenotypic confirmation of the *eIF(iso)4E* edited and wild type plants after 30 days of inoculation. Edited and wild type plants were grown in a growth chamber. A) Phenotypic comparison of edited and wild type plants. B) Phenotype for TuMV symptoms in edited and WT plants. Left, edited plants; right, wild type plants.

Kim et al. [18] have developed transgenic Chinese cabbage by targeting *eIF(iso)4E* at the protein level to develop broad-spectrum resistance against potyviruses, such as TuMV (Turnip mosaic virus). TuMV particle accumulation was abridged when *eIF(iso)4E* proteins mutated in these amino acids were over-expressed in transgenic plants. Furthermore, reduced reciprocity between *eIF(iso)4E* and TuMV VPg was shown by the results to potentially cause conferred resistance [18]. However, the resistance mechanism remained unclear and required further studies to mutate *eIF4E* in Chinese cabbage and to study the variations at the DNA sequence level. Consequently, we evaluated our *eIF(iso)4E* edited T_1_ plants for TuMV resistance to the potyvirus (Fig. 4). Genome-edited plants harboring mutations in *eIF(iso)4E* conferred resistance against TuMV. We showed that there was a strong negative correlation (*r = −0.938*) between gene editing efficiency related to indel frequency and TuMV resistance evaluation using the ELISA score (Fig. 5). Additionally, we confirmed that homozygous lines show uniform resistance to TuMV resistance in an LMO glass house (Fig. 6). While no significant phenotypic difference was observed between the *eIF(iso)4E*-edited plants and wild type plants under our growth experimental conditions, it is possible that the mutation in the *eIF(iso)4E* gene could have adverse effect on growth of Chinese cabbage under different stress conditions.

Recently, genome editing in Chinese cabbage has been performed for different traits, such as FLOWERING LOCUS C (FLC) for early flowering, *BrVRN1* gene for delayed flowering, and *PR55/B* gene for self-incompatibility [41–43]. Moreover, DNA-free (transgene-free) genome editing by targeting the vernalization determinant FRIGIDA and phytoene desaturase gene (FRI and PDS) genes has also been done to pave toward minimizing GMO legislation-related issues [34]. However, no CRISPR/Cas9-based genome editing study has been conducted to achieve viral resistance in Chinese cabbage. In the current study, we applied CRISPR/Cas9 gene editing to induce mutations in the Chinese cabbage *eIF(iso)4E* gene by *Agrobacterium*-mediated transformation. Different indel patterns of mutations in the *eIF(iso)4E* gene, including mosaic, double, and single, were detected and verified by sequencing in T_0_ and subsequent T_1_ generations. Our work demonstrated effective CRISPR/Cas9 editing of Chinese cabbage *eIF4E1*, triggering resistance to potyvirus. The findings of the current study can be practically extended to the breeding programs of relative *Brassica* spp. and will help develop virus-resistant cultivars. Hence, targeted genome editing could be anticipated to expedite plant breeding for disease resistance, particularly for virus resistance in crop plants, by enabling the incorporation of precise and expected genetic modifications directly into an elite cultivars background.

## Materials and methods

### Plant materials and conditions for growth in vitro

The Chinese cabbage cultivar, “Seoul” (Dong-bu Seed, Korea), which has been reported to be susceptible to turnip mosaic virus [18], was used for *Agrobacterium*-mediated transformation. The seeds used in this study were first disinfected through seed coat sterilization in 70% ethanol for 1 to 2 min, followed by shaking in 10% commercial Clorox for 10 to 15 min at 100 rpm. The seeds were thoroughly rinsed five to six times with sterilized water. The sterilized seeds were germinated on 1/2 MS medium [44] supplemented with 2% sucrose and 0.8% agar. These seeds were subjected to *in vitro* cultivation to grow hypocotyls up to 6–8 cm for five days at 25°C. The hypocotyls were cut, avoiding the apical meristem and quickly cut into 7–8 mm segment intervals before the first true leave emerged. Forty explants were cultured in a 15 x 90 mm petri dish containing 0.8% MS medium (pH 5.8) supplemented with 3% sucrose, BA (4 mg/ml), NAA (1 mg/ml). All of the cultures were incubated in the controlled incubation room for 3 days at 25°C under dark condition. These explants were used for tissue culture and *Agrobacterium*-mediated transformation.

### Construction of single guide RNAs (sgRNAs) and CRISPR/Cas9 vector

The sequences of *eIF(iso)4E*, which have been the Chinese cabbage potyvirus, as previously reported by Jenner et al. [35], were downloaded from the NCBI (http://www.ncbi.nlm.nih.gov/) (Table 1). The BLAST searches were performed using the Brassica database (BRAD, http://brassicadb.cn/) to obtain sequences around *eIF(iso)4E* in *B. rapa*. The *eIF(iso)4E* genes (*Bra035531*, *Bra039484*, and *Bra035393*) of Chinese cabbage were aligned and selected as the target sequences (Fig. 1A). The CRISPR RGEN tools (http://www.rgeneome.net) was used to identify appropriate target sgRNA sequences and designed sgRNAs common for each Chinese cabbage *eIF(iso)4E* gene (Fig. 1B). All three sgRNAs (sgRNA1, 2, and 3) were designed to target *Bra035531, Bra035393* and *Bra039484* genes (Fig. 1A). To express Cas9 protein in plants, the maize codon-optimized Cas9 driven by the CaMV 35S promoter. The pECO101 vector with the Cas9 gene was cloned into a binary vector under the control of the AtU6 promoter (Fig 1B). The sgRNAs scaffold was annealed and cloned into a *BsaI* restriction site in the vector pECO101 by Golden-Gate cloning according to a previously reported method [45]. The resulting CRISPR/Cas9 vectors were transformed into *Agrobacterium tumefaciens* strain GV3101 by heat shock.

**Figure 7:**
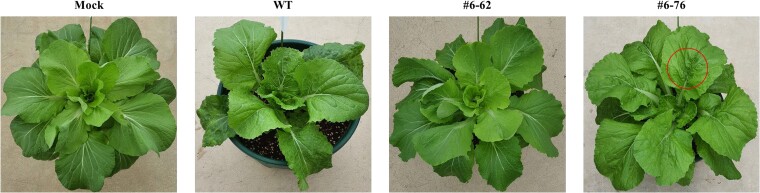
Phenotypic confirmation of the *eIF(iso)4E* edited plants after 30 days of planting a pot. Mock, WT and edited plants (#6–62 and #6–76) were grown in an LMO glass house. The red circle indicates mosaic, which is one of the symptoms of TuMV.

### *Agrobacterium*-mediated transformation of Chinese cabbage

A single colony of *Agrobacterium* was grown in 10 mL of liquid YEP medium having 50 mg/mL spectinomycin at 28°C until it reached an OD_600_ value of 0.6–0.8. The pellet was harvested by centrifuge at 13000 rpm for 15 min at 4°C using *Agrobacterium* suspension.. The gained pellet was re-suspended entirely in liquid 1/2 MS medium (pH 5.2) containing 3.6% glucose. The *Agrobacterium* re-suspension was applied to infect hypocotyl explants and co-cultured for 1 h, after infection, they were followed by transfer to sterile filter paper to clear remained suspension completely. For co-culture with *agrobacterium*, these explants were placed on the MS medium containing 3% sucrose, BA (4 mg/ml), NAA (1 mg/ml), and 0.8% plant agar (pH 5.2) and incubated in two days at 22°C and dark condition. The co-cultured explants were transferred to the MS selection medium containing 3% sucrose, AgNO_3_ (4 mg/L), IBA (4 mg/L), NAA (3 mg/L), hygromycin (10 mg/L), and 0.8% plant agar (pH 5.6). The number of calli and shoots formed on the explants was determined after cultivation for three and 10 weeks, respectively. Finally, the regenerated shoots were transferred to a rooting medium containing 1/2 MS medium, 3% sucrose, and 0.5% plant agar (pH 5.8). The regenerated plants were cultivated in a growth room maintained at 22°C and a 16 h light/8 h dark cycle.

### DNA extraction and PCR analysis

Total genomic DNA (gDNA) was extracted from the leaf samples of potential transgenic plants using the cetyltrimethylammonium bromide (CTAB) method [46]. gDNA was enumerated using a Nanodrop spectrophotometer (Nanodrop Technology, Inc., Wilmington, DE, USA) and diluted to 50 ng/mL. Transgenic plants were evaluated by PCR analysis using HPT (Hygromycin phosphotransferase) and Cas9 gene-specific primers **(**Table 1**)**. The amplification settings was adjusted as follows: pre-denaturation at 95°C for 10 min, followed by 35 cycles of 95°C for 30 s, 58°C for 30 s, 72°C 30 s, and final extension at 72°C for 10 min.

### Target deep-sequencing and mutation detection

Plant that are transgenic were assessed for mutation identification using specific target primers linked to sgRNA target regions (Table 1). The samples detected with target site insertion were subjected to deep sequence analysis. For the deep-sequencing analysis, three rounds of PCR were performed, and during the first PCR, analysis primers were designed with a amplication size of 600–800 bp. PCR was performed with a final volume of 20 μL, containing 50 ng template DNA concentration, 1.0 pmol of reverse and forward primers, 10 mM dNTPs, 10x Hipi buffer, and 0.2 units of Hipi plus Taq polymerase. The following protocol was used for PCR amplification: 95°C preheating for 3 min and 95°C for 30 s, followed by 10 cycles of 72°C for 30 s, 72°C for 45 s, and 20–30 cycles at 95°C for 30 s, 62°C for 30 s, 72°C for 45 s, and a final extension of 5 min at 72°C. The second round of PCR was used to attach the adapter, and the primers were designed with a product size of 150–250 bp. The cleaned PCR products were subjected to index amplification with known barcodes. Next, index PCR was performed using a dual indexing and adapter kit (Illumina, California, USA). Finally, the PCR products were purified using a PCR clean-up kit (Cosmo Genetech, Seoul, Korea). The refined PCR products were sequenced directly according to the method by Shokralla et al. [47].

### Inoculations of the TuMV virus

Frozen TuMV stock stored at −80°C was used to prepare the virus inoculum. A preliminary experiment was conducted by inoculating 3-week-old plants of susceptible Chinese cabbage to confirm the virulence of the strain. Briefly, frozen inoculate was crushed in 0.1 M potassium phosphate buffer (pH 7.0), combined with 400-grit carborundum, and scrubbed on the lower leaves of susceptible plants. Subsequently, the leaves were washed with distilled water after 10–20 min of inoculation [48]. The symptomatic leaves showing typical TuMV symptoms were harvested four weeks after inoculation and double checked with a double-antibody sandwich enzyme-linked immunosorbent assay (DAS-ELISA) test kit (Agdia Inc., Elkhart, IN, USA) for the virus presence. The putative genome-edited plants and susceptible controls were inoculated, as described above. In detail, Chinese cabbage plants with 2–4 true leaves were used for viral inoculation and each plant one pair cotyledons were inoculated. The inoculated and non-inoculated control plants (mock) were grown in a growth chamber at 26°C under 16 h/8 h light/dark conditions in white fluorescent light.

### Evaluation of resistance to turnip mosaic viruses

The inoculated plants were regularly inspected for the appearance of symptoms after viral inoculation. Leaves were observed developing typical TuMV symptoms [18] to discriminate resistant and susceptible plants after seven days of viral inoculation. The leaf tissues were subjected to DAS-ELISA to evaluate the presence of the virus. TuMV viral accumulation was finally assessed 21 days after inoculation. DAS-ELISA was conducted to identify the accretion of the coat protein of TuMV. Three replicates of inoculated and upper non-inoculated leaves of T_1_ lines were assessed for the ELISA test. The absorbance value of each sample was measured at 405 nm using an ELISA reader (Titertek, Huntsville, AI, USA). The statistical significance of the data was determined with a t-test using the R Studio v. 4.0.3 package.

## Acknowledgments

This work was carried out with the support of “Cooperative Research Program for Agriculture Science and Technology Development (Project No. PJ01652201)” Rural Development Administration, Republic of Korea.

## Credit authorship contribution

**Ye-Rin Lee:** Conceptualization, Investigation, Formal analysis, Methodology, Software, Writing - original draft, Writing – review & editing. **Muhammad Irfan Siddique:** Investigation, Writing - original draft, Writing – review & editing **Do-Sun Kim:** Data curation, Formal analysis, Writing – review & editing. **Eun Su Lee:** Data curation, Formal analysis. **Koeun Han:** Data curation, Formal analysis. **Sang-Gyu Kim:** Formal analysis, Writing – review & editing. **Hye-Eun Lee:** Funding acquisition, Project Administration, Supervision, Validation, Writing – review & editing.

## Data availability

The data and materials used to support the findings of this study are available from the corresponding author upon request.

## Conflict of interest statement

None delcared.

## Supplementary data

Supplementary data is available at *Horticulture Research* online.

## Supplementary Material

Web_Material_uhad078
