# Supplementary material for: CRISPR/Cas9-mediated gene editing to confer turnip mosaic virus (TuMV) resistance in Chinese cabbage (Brassica rapa)
Source: Hortic Res. 2023 Apr 21;10(6):uhad078. doi: 10.1093/hr/uhad078 (PMC10261878; doi:10.1093/hr/uhad078)
Supplement: Web_Material_uhad078 [file web_material_uhad078.zip › 2022-11-22-supply file ver1.pptx]

## Slide 1
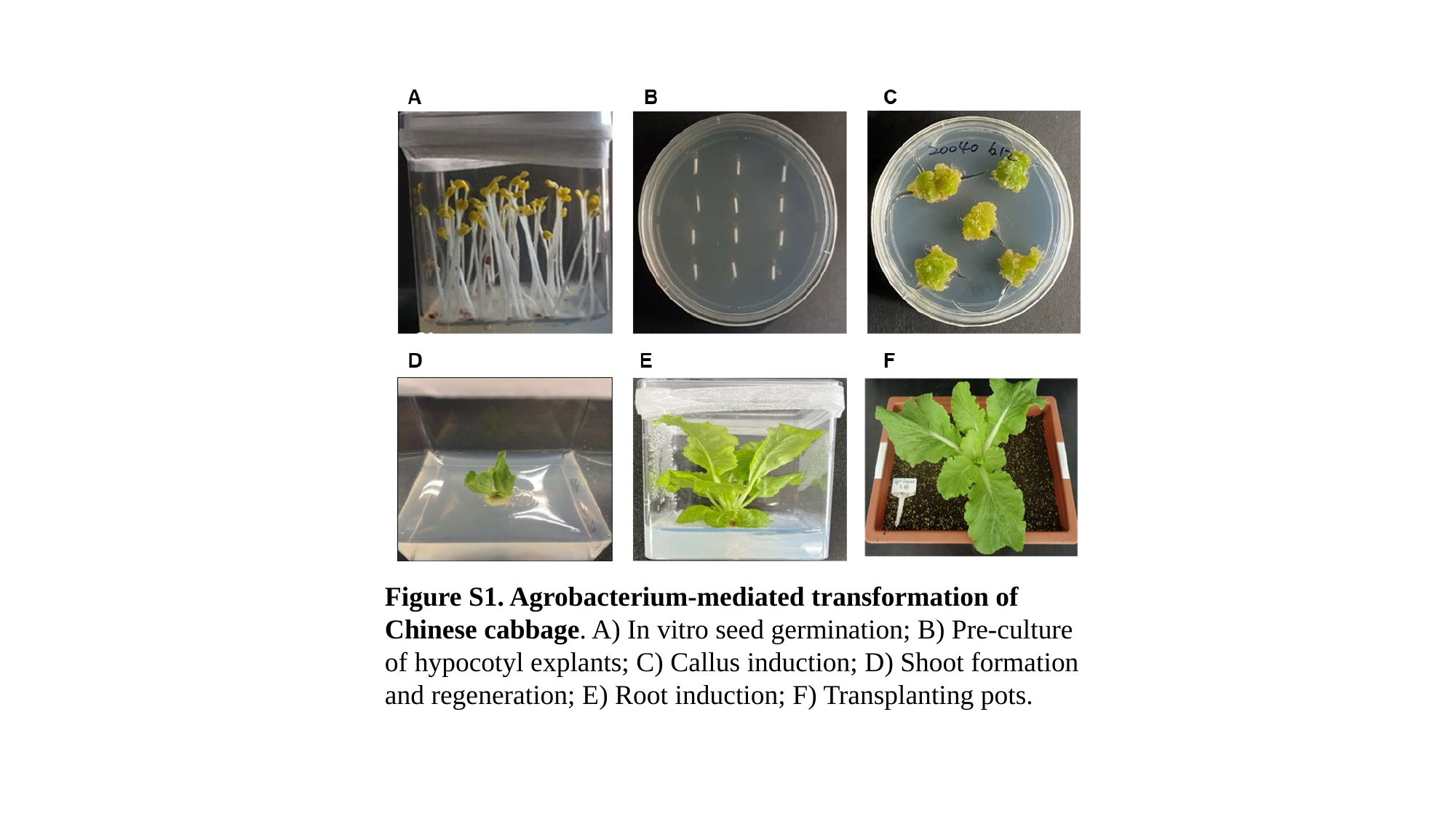

Figure S1. Agrobacterium-mediated transformation of Chinese cabbage. A) In vitro seed germination; B) Pre-culture of hypocotyl explants; C) Callus induction; D) Shoot formation and regeneration; E) Root induction; F) Transplanting pots.

## Slide 2
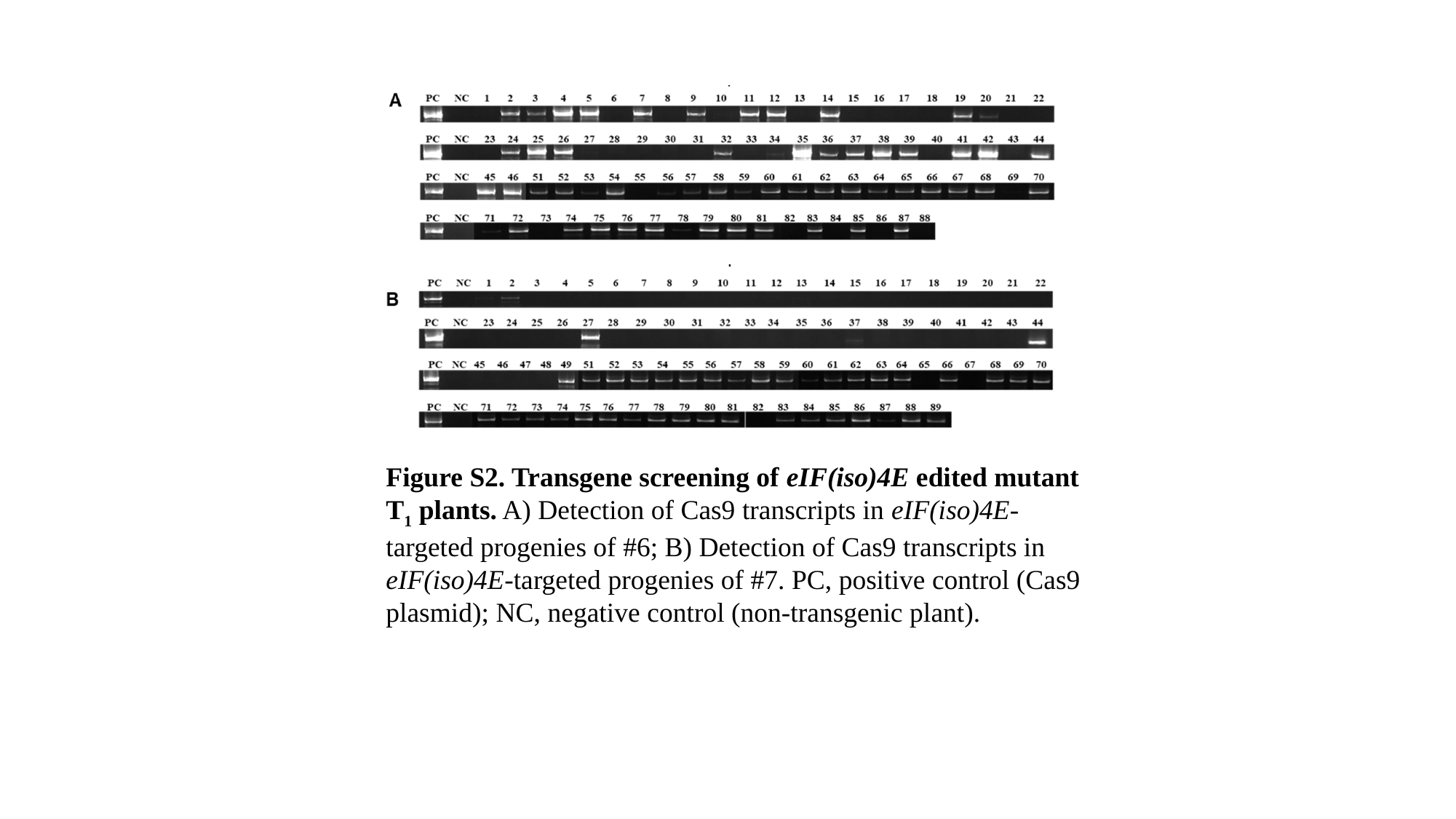

Figure S2. Transgene screening of eIF(iso)4E edited mutant T1 plants. A) Detection of Cas9 transcripts in eIF(iso)4E-targeted progenies of #6; B) Detection of Cas9 transcripts in eIF(iso)4E-targeted progenies of #7. PC, positive control (Cas9 plasmid); NC, negative control (non-transgenic plant).

## Slide 3
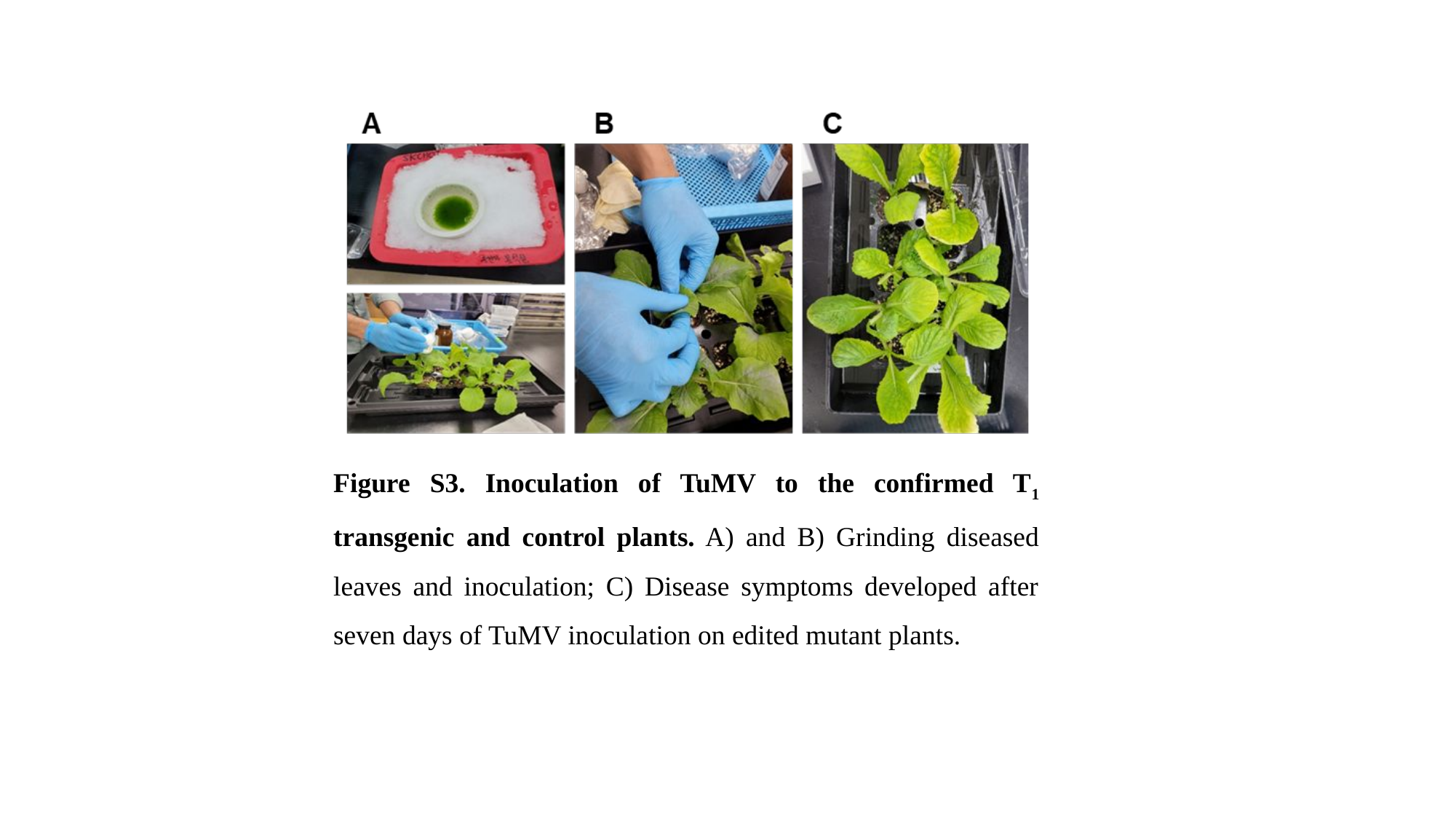

Figure S3. Inoculation of TuMV to the confirmed T1 transgenic and control plants. A) and B) Grinding diseased leaves and inoculation; C) Disease symptoms developed after seven days of TuMV inoculation on edited mutant plants.
